# Ecotoxicological Impacts of Microplastics and Cadmium Pollution on Wheat Seedlings

**DOI:** 10.3390/nano16020090

**Published:** 2026-01-09

**Authors:** Shuailing Yang, Steven Xu, Tianci Guo, Zhangdong Wei, Xingchen Fan, Shuyu Liang, Lin Wang

**Affiliations:** 1 Miami College, Jinming Campus, Henan University, Kaifeng 475004, China; lyzy8570@163.com (S.Y.); gtc5342@henu.edu.cn (T.G.); 18063388582@163.com (X.F.); 19836887018@163.com (S.L.); 2 Thermo Fischer Scientific-PPD Bioanalytical Laboratory Address, 3230 Deming Way, Middleton, WI 53563-1475, USA; scx1@scarletmail.rutgers.edu; 3 College of Geographical Sciences, Faculty of Geographical Science and Engineering, Henan University, Zhengzhou 450046, China

**Keywords:** polyethylene microplastics, cadmium, antioxidant enzymes, peroxidase, bioaccumulation, ecotoxicology

## Abstract

As plastic and heavy metal pollution continue to escalate, the co-occurrence of microplastics and heavy metals in the environment poses significant threats to ecosystems and human health. This study was designed to explore the combined effects of polyethylene microplastics (PE-MPs) and cadmium (Cd) pollution on wheat seedlings, focusing on antioxidant enzyme activity and Cd bioaccumulation. At low concentrations of PE ( $1\;\mathrm{mg}\cdot L^{-1}$ ), peroxidase (POD) activity in wheat shoots slightly increased without significance, while at higher concentrations ( $50\;\mathrm{mg}\cdot L^{-1}$ and $100\;\mathrm{mg}\cdot L^{-1}$ ) of PE, POD activity was significantly inhibited compared to $0\;\mathrm{mg}\cdot L^{-1}$ PE treatment. At Cd exposure activity, with POD activity in the shoots increasing by 73.7% at $50\;\mu \mathrm{mol}\cdot L^{-1}$ ${\mathrm{Cd}}^{2+}$ compared to $0\;\mu \mathrm{mol}\cdot L^{-1}$ Cd treatment. When wheat seedlings were exposed to a combination of $50\;\mathrm{mg}\cdot L^{-1}$ PE and Cd at different concentrations Cd, significant differences in POD activity were observed in the shoots compared to the control group, showing an upward trend with increasing Cd concentration. However, the addition of PE suspension generally reduced POD activity in wheat shoots compared to Cd treatment alone. Specifically, the presence of $50\;\mathrm{mg}\cdot L^{-1}$ PE did not significantly alter POD activity in the wheat shoots ( p>0.05 ). Furthermore, exposure to different concentrations of Cd resulted in a general increase in POD activity of roots, with significant differences observed at $5\;\mu \mathrm{mol}\cdot L^{-1}$ and $25\;\mu \mathrm{mol}\cdot L^{-1}$ Cd ( p<0.05 ). Regarding Cd bioaccumulation, at Cd low concentrations ( $1\;\mu \mathrm{mol}\cdot L^{-1}$ and $5\;\mu \mathrm{mol}\cdot L^{-1}$ ), PE significantly promoted Cd accumulation in the shoots. However, at high Cd concentrations ( $50\;\mu \mathrm{mol}\cdot L^{-1}$ ), PE microplastics reduced Cd accumulation in the shoots but promoted its accumulation in the roots.These results suggest that PE microplastics influence the bioavailability of Cd, mitigating the toxic effects of high Cd concentrations. This paper scientifically elucidates the ecotoxicological effects of co-contamination for microplastics and heavy metals, also their potential impacts on agricultural production are discussed.

## 1. Introduction

Plastics are among the most widely produced synthetic materials globally, valued for their low cost, versatility, and corrosion resistance. They have become integral to modern life, with widespread applications in agriculture, manufacturing, healthcare, and domestic products. However, the uncontrolled disposal and long-term persistence of plastics have led to their widespread accumulation in natural environments. Over time, plastics undergo fragmentation into smaller particles under ultraviolet radiation, oxidation, and physical weathering, forming microplastics (MPs), which are usually defined as plastic particles because its diameter is smaller than 5 mm. These particles are now ubiquitous across marine, freshwater, and terrestrial ecosystems and can persist for centuries due to their resistance to biodegradation.

**Figure 1:**
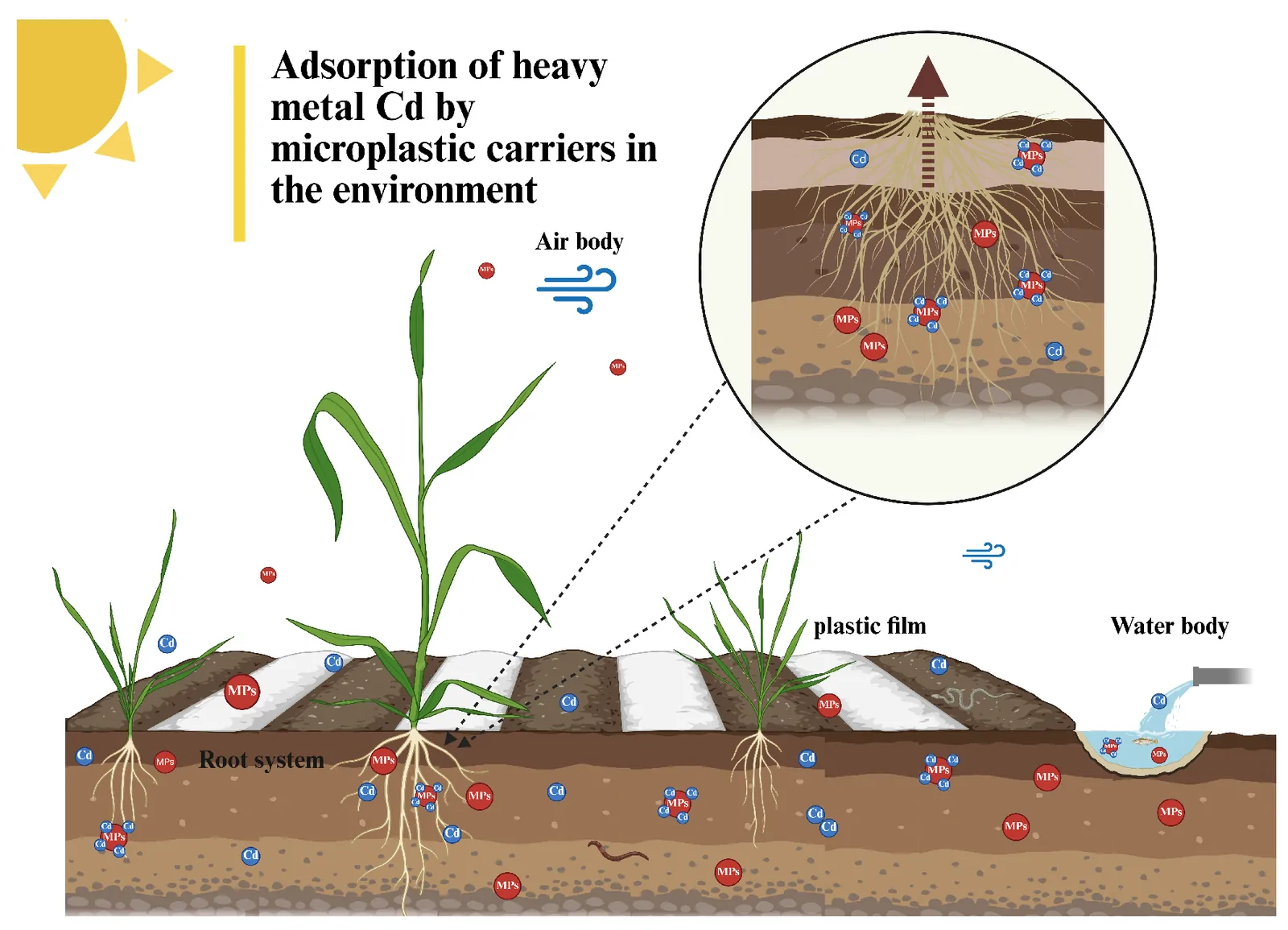
Microplastic-mediated adsorption of heavy metal Cd in the environment.

Microplastics in soil environments are currently receiving increasing attention. Microplastic concentrations in industrial park soils are significantly higher than those in urban and rural areas, while urban soils contain levels one order of magnitude higher than agricultural soils [1]. Due to their small particle size, large surface area, and strong hydrophobicity, MPs possess exceptional adsorption capabilities for organic and inorganic contaminants (Figure 1). MPs can be consumed by various organisms, transferred across food chains, and even detected in human tissues such as blood and breast milk [2,3,4,5,6], posing significant ecological and health risks. Recent studies have confirmed that MPs can be absorbed by plant roots and translocated to aboveground tissues, altering root morphology, biomass accumulation, photosynthetic activity, and nutrient uptake. While the physiological effects of MPs on plants appear to depend on particle type, size, and concentration, their overall presence disrupts normal metabolic and antioxidative processes.Current research indicates that microplastics influence plant growth through multiple mechanisms. For instance, polystyrene microplastics reduce rice photosynthetic rates and chlorophyll content by regulating carbon metabolism and lignin production via genes such as *OsOFP2* [7]. In soybeans, they inhibit the production of L-tryptophan and salicylic acid 2-O- β -glucoside [8]. For wheat, it has been demonstrated that polystyrene microplastics can alter the plant’s carbon cycle.

At the same time, heavy metal pollution has emerged as one of the most critical environmental issues globally. Among the heavy metals, cadmium (Cd) is of particular concern due to its high toxicity, mobility, and bioaccumulative nature. Cd enters the environment through mining, industrial effluents, fertilizers, and waste incineration, where it cannot be biologically degraded. In plants, Cd interferes with essential nutrient uptake, induces reactive oxygen species (ROS) accumulation [9], and damages cellular membranes and organelles. In humans, Cd exposure has been linked to various diseases, including renal dysfunction, bone damage, and cardiovascular disorders. In China, Cd contamination in agricultural soils has become a major challenge, with large areas of farmland exceeding national safety limits, posing direct risks to food quality and public health [10,11,12].

In recent years, the co-occurrence of microplastics and heavy metals in environmental matrices has garnered increasing attention [13]. Microplastics may serve as carriers or sinks for heavy metals [14], thereby altering their bioavailability, transport behavior, and toxic effects in biotic systems [15]. However, the interactive mechanisms between MPs and heavy metals in terrestrial plants remain poorly understood. Studies suggest that MPs may enhance metal uptake at low concentrations through adsorption and carrier effects, while at higher concentrations, they may compete with metal ions, reducing their bioavailability. Such complex interactions can influence plant antioxidant defense systems, disturb redox homeostasis, and alter metal accumulation patterns within plant tissues. The dualistic role of MPs in these interactions highlights the need for further investigation into whether they exacerbate or mitigate heavy metal toxicity under combined exposure conditions.

Wheat (*Triticum aestivum* L.), a world staple food crop, is of particular significance for food security, especially in countries like China, where it sustains a large portion of the population. Therefore, understanding the response mechanisms of wheat when simultaneously exposed to microplastics and cadmium is crucial for assessing potential ecological risks and food safety concerns. This study is designed to explore the effects of polyethylene microplastics (PE-MPs), cadmium (Cd) and their mixtures on antioxidant enzyme activity, metal accumulation, and nutrient balance in hydroponic wheat seedlings. The findings aim to elucidate the mechanisms by which PE-MPs influence Cd toxicity and uptake in plants, providing a scientific foundation for assessing the ecological risks associated with microplastic–heavy metal interactions in terrestrial agroecosystems.

## 2. Materials and Methods

### 2.1. Experimental Design

This experiment was conducted in two phases. The first phase was designed to characterize the effects of different concentrations of PE-MPs on wheat growth. Based on the results of the first phase, the second phase was designed to examine the efforts of combined PE-MPs and Cd contamination on wheat growth.

**Figure 2:**
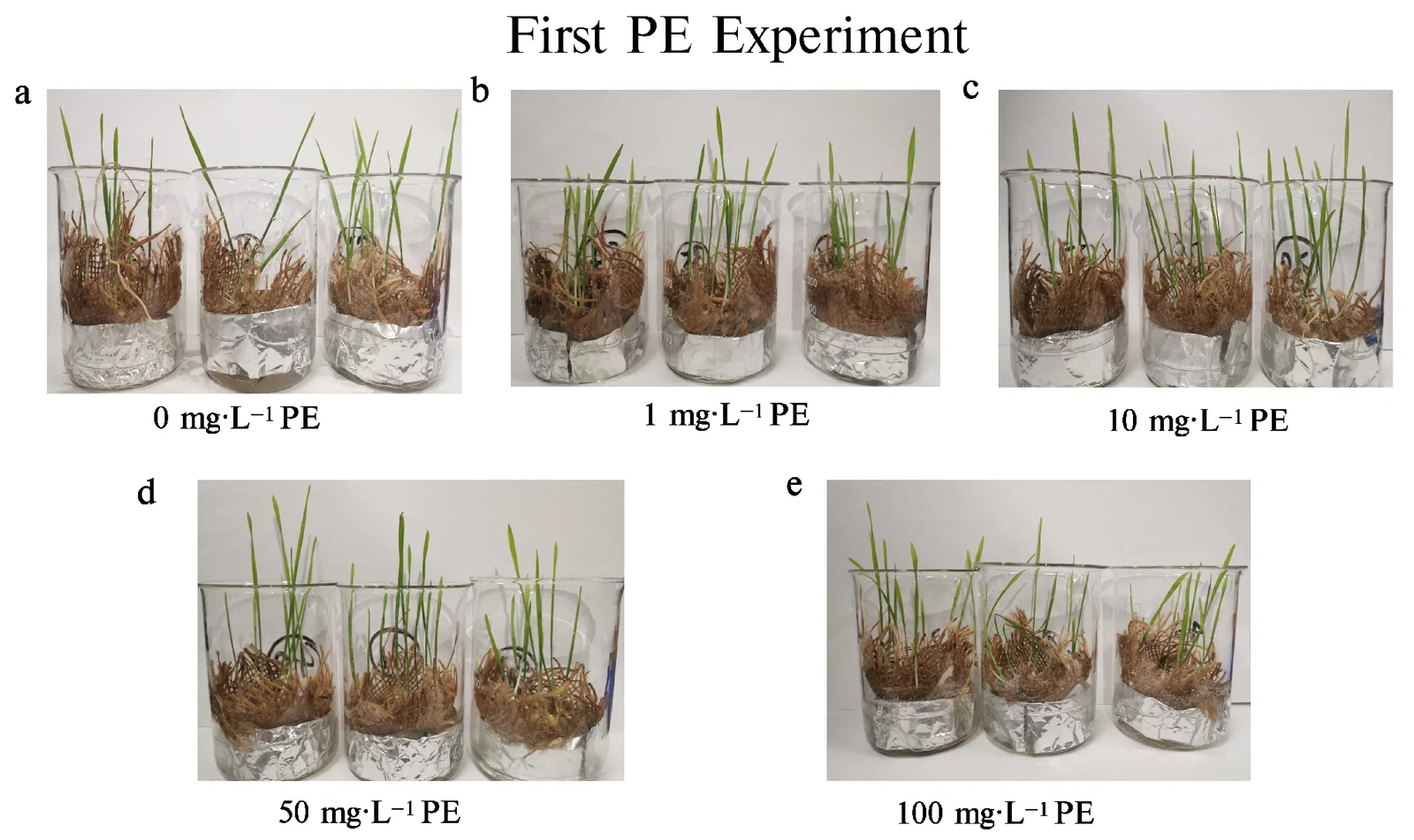
Effect of different concentrations of PE-MPs on wheat growth. (**a**) $0\;\mathrm{mg}\cdot L^{-1}\;\mathrm{PE}$ ; (**b**) $1\;\mathrm{mg}\cdot L^{-1}\;\mathrm{PE}$ ; (**c**) $10\;\mathrm{mg}\cdot L^{-1}\;\mathrm{PE}$ ; (**d**) $10\;\mathrm{mg}\cdot L^{-1}\;\mathrm{PE}$ ; (**e**) $100\;\mathrm{mg}\cdot L^{-1}\;\mathrm{PE}$ .

Phase 1 of the experiment aimed to find the impact of different concentrations of PE-MPs on wheat growth (Figure 2). Wheat seeds (Dwarf Resistant 58, purchased from Shaanxi Changfeng Seed Industry Co., Ltd.) were surface-sterilized by soaking in a 5% (*v*/*v*) sodium hypochlorite solution for 25 min, after which they were thoroughly rinsed with deionized water. After sterilization, the seeds were placed on filter paper in 9cm petri dishes and incubated at 20 °C with 80% humidity. The culture solution was replaced every 48 h to maintain a consistent concentration in the containers. After 48 h, uniform and healthy seedlings with germinating roots were selected and used for the experiment. Seedlings were incubated in a constant-temperature chamber (Jinghong Geothermal Incubator of Shanghai, GNP-9160) at 25 °C and 75% humidity, with a light intensity of 2600lx and a light/dark cycle of 16 h/8 h. The root systems were fixed on coarse linen and transferred to 500mL beakers containing the culture solution, with 10 seedlings per beaker. The beakers were then placed in a temperature-controlled incubator for continued growth.

The concentrations of PE-MPs were set at $0\;\mathrm{mg}\cdot L^{-1}$ , $1\;\mathrm{mg}\cdot L^{-1}$ , $10\;\mathrm{mg}\cdot L^{-1}$ , $50\;\mathrm{mg}\cdot L^{-1}$ , and $100\;\mathrm{mg}\cdot L^{-1}$ , with three replicates for each treatment. Sampling began at 7:00 a.m. on day 8, and measurements were taken for the dry weight and peroxidase (POD) enzyme activity of both the shoot and root systems.

The PE-MPs solution was prepared by 10μm powdered microplastics (purchased from Guanghong Polymer Materials Co., Dongguan, China) at weight of 0,1,10,50,100mg , which were then mixed into 50mL of deionized water to form the PE-MPs suspensions. Then, we can replace the suspension with the same concentration every 48 h to maintain a consistent growth environment for the wheat.

**Figure 3:**
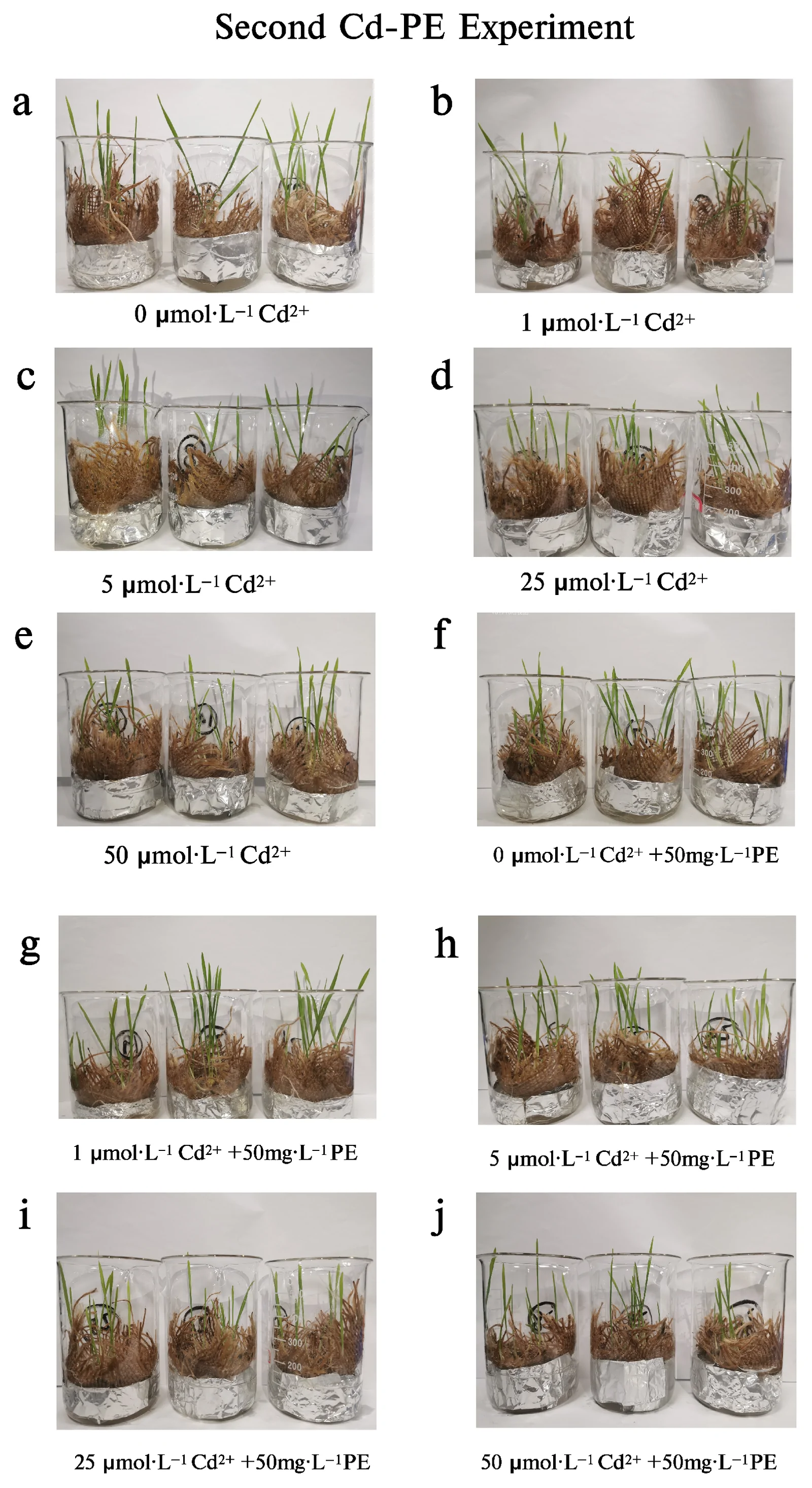
Effect of PE-MPs and Cd on wheat growth. (**a**) $0\;\mu \mathrm{mol}\cdot L^{-1}\;{\mathrm{Cd}}^{2+}$ ; (**b**) $1\;\mu \mathrm{mol}\cdot L^{-1}\;{\mathrm{Cd}}^{2+}$ ; (**c**) $5\;\mu \mathrm{mol}\cdot L^{-1}\;{\mathrm{Cd}}^{2+}$ ; (**d**) $25\;\mu \mathrm{mol}\cdot L^{-1}\;{\mathrm{Cd}}^{2+}$ ; (**e**) $50\;\mu \mathrm{mol}\cdot L^{-1}\;{\mathrm{Cd}}^{2+}$ ; (**f**) $0\;\mu \mathrm{mol}\cdot L^{-1}\;{\mathrm{Cd}}^{2+}+50\;\mathrm{mg}\cdot L^{-1}\;\mathrm{PE}$ ; (**g**) $1\;\mu \mathrm{mol}\cdot L^{-1}\;{\mathrm{Cd}}^{2+}+50\;\mathrm{mg}\cdot L^{-1}\;\mathrm{PE}$ ; (**h**) $5\;\mu \mathrm{mol}\cdot L^{-1}\;{\mathrm{Cd}}^{2+}+50\;\mathrm{mg}\cdot L^{-1}\;\mathrm{PE}$ ; (**i**) $25\;\mu \mathrm{mol}\cdot L^{-1}\;{\mathrm{Cd}}^{2+}+50\;\mathrm{mg}\cdot L^{-1}\;\mathrm{PE}$ ; (**j**) $50\;\mu \mathrm{mol}\cdot L^{-1}\;{\mathrm{Cd}}^{2+}+50\;\mathrm{mg}\cdot L^{-1}\;\mathrm{PE}$ .

In the second experiment phase, PE-MPs at a concentration of $50\;\mathrm{mg}\cdot L^{-1}$ was combined with different concentrations of Cd to assess the effect of composite contamination (Figure 3). A total of 10 treatment groups were set up: $0,\;1,\;5,\;25,\;50\;\mu \mathrm{mol}\cdot L^{-1}\;{\mathrm{Cd}}^{2+}$ , $50\;\mathrm{mg}\cdot L^{-1}\;\mathrm{PE}+0,\;1,\;5,\;25,\;50\;\mu \mathrm{mol}\cdot L^{-1}\;{\mathrm{Cd}}^{2+}$ . All treatments had three replicates, and samples were collected at 7:00 am on day 8. The dry weight, peroxidase (POD) enzyme activity and Cd concentration in both the shoots and roots of wheat were measured.

The Cd solution was prepared by dissolving 50, 250, 1250, and 2500μL of ${\mathrm{CdCl}}_{2}\cdot 2.5H_{2}O$ (analytical grade) in 50mL of deionized water to create stock solutions with Cd concentrations of 0, 1, 5, 25, and $50\;\mu \mathrm{mol}\cdot L^{-1}$ . For the PE+Cd composite solutions, 2.5mg of PE-MPs were mixed into the Cd solutions to achieve the following gradient concentrations: $0\;\mu \mathrm{mol}\cdot L^{-1}\;{\mathrm{Cd}}^{2+}+50\;\mathrm{mg}\cdot L^{-1}\;\mathrm{PE}$ , $1\;\mu \mathrm{mol}\cdot L^{-1}\;{\mathrm{Cd}}^{2+}+50\;\mathrm{mg}\cdot L^{-1}\;\mathrm{PE}$ , $5\;\mu \mathrm{mol}\cdot L^{-1}\;{\mathrm{Cd}}^{2+}+50\;\mathrm{mg}\cdot L^{-1}\;\mathrm{PE}$ , $25\;\mu \mathrm{mol}\cdot L^{-1}\;{\mathrm{Cd}}^{2+}+50\;\mathrm{mg}\cdot L^{-1}\;\mathrm{PE}$ , and $50\;\mu \mathrm{mol}\cdot L^{-1}\;{\mathrm{Cd}}^{2+}+50\;\mathrm{mg}\cdot L^{-1}\;\mathrm{PE}$ . Similarly, the suspension was replaced with the same concentration every 48 h to maintain a consistent growth environment for the wheat.

### 2.2. Experimental Methods

#### 2.2.1. Peroxidase Activity Measurement

The peroxidase (POD) enzyme activity was measured using a commercial assay kit (Beijing Solarbio Science & Technology Co., Ltd., Beijing, China). Fresh wheat shoots and roots were carefully washed with ultra-pure water, and 0.10g of each sample was blended with 1mL of pre-chilled enzyme extraction solution. A homogenate was centrifuged at 8500rpm for 10min at 4 °C. The supernatant was separated and stored on ice for subsequent analysis. POD activity was measured using a visible spectrophotometric method with a 722N UV/VIS spectrophotometer (Shanghai INESA Scientific Instrument, Shanghai, China). In a 1mL cuvette, the reaction mixture contained 15μL of homogenate, 270μL of distilled water, 520μL of reagent 1, 130μL of reagent 2 working solution, and 135μL of reagent 3. Absorbance was recorded at 470nm , and the absorbance change ( ΔA ) is calculated using the following formula:

(1) $$\Delta A=A_{2}-A_{1}.$$

The POD activity ( U/g ) was then calculated using the following formula:

(2) $$\mathrm{POD}\;\mathrm{Activity}\;\left(U/g\right)=\frac{7133\times \Delta A}{W},$$

where *W* represents the weight of the sample ( g ).

#### 2.2.2. Cd Element Content Measurement

For Cd concentration determination, three plants from each treatment were selected. The shoots and roots were dried in an oven at 75 °C for 48 h to constant weight, then ground into powder. Each dried sample was placed into a Teflon digestion tube, and 10mL of a ${\mathrm{HNO}}_{3}$ : ${\mathrm{HClO}}_{4}$ (4:1, *v*/*v*) mixture was added. The tubes were left overnight and then heated in a microwave digestion system until the solution became clear and colorless. After removing the acid through the above procedure, the solution was transferred to a 50mL volumetric flask, and the volume was made up to 50mL with 2% (*v*/*v*) ${\mathrm{HNO}}_{3}$ . After filtration through a 0.45μm filter, the samples were analyzed for Cd content using an inductively coupled plasma mass spectrometer (ICP-MS, Elan DRC-e, PerkinElmer, USA). The same digestion and analytical methods were used for wheat samples and national certified reference material (CRM) corn (GB1353, Beijing, China) to determine the recovery rate of Cd in plant samples. The element recovery rate of the ICP-MS analytical method ranges from 95% to 105% .

### 2.3. Statistical Analysis

All results are shown as mean values ( n=3 ) ± standard deviation (SD). The statistical analyses were conducted using SPSS Statistics 22 (IBM, USA). Normality of the data was assessed using the Shapiro-Wilk test, and homogeneity of variance was tested using Levene’s test. One-way analysis of variance (ANOVA) was conducted, and Tukey’s Honest Significant Difference (HSD) test was used to evaluate significant differences between treatment groups. Based on results from the first phase, an appropriate PE-MPs concentration was selected for subsequent analysis of composite pollution with Cd. A *p*-value <0.05 was considered statistically significant, and differences were denoted by different letters. All figures were created using Origin 2025 software (Origin Labs, USA).

## 3. Results and Discussion

### 3.1. Effects of PE-MPs on Wheat Seedlings

#### 3.1.1. Effects of PE-MPs on the Biomass in Wheat Seedlings

In the treatment with PE-MPs suspension alone (Table 1), the overall dry weight of wheat shoots was higher than that of the $0\;\mathrm{mg}\cdot L^{-1}$ PE treatment, though no significant differences were observed. Similarly, the dry weight of the root systems was reduced compared to the $0\;\mathrm{mg}\cdot L^{-1}$ PE treatment, although this difference was not statistically significant. These findings suggest that while PE suspension did not significantly affect the biomass in either the root or shoot systems, there may be a slight trend toward a reduction in root biomass. This may be because the root system is the first part of the plant to come into direct contact with microplastics, which may restrict their growth.

It is known that when microplastics, particularly at the micron and nanometer scale, enter plant roots and accumulate within the tissues, they can suppress [16], leading to a decrease in overall biomass. This suppression of photosynthetic activity can result in reduced dry weight accumulation, particularly in the root systems. However, in this experiment, the lack of significant differences suggests that the PE concentrations tested may not have been sufficient to cause observable growth inhibition under the given conditions.

#### 3.1.2. Effects of PE-MPs on the Peroxidase Activity in Wheat Seedlings

Peroxidase (POD) is a crucial antioxidant enzyme widely distributed in plant tissues, playing a key role in mitigating oxidative stress. It catalyzes the decomposition of $H_{2}O_{2}$ and the oxidation of phenolic and amine compounds. As such, POD serves a dual function: it decomposes hydrogen peroxide generated from the dismutation of superoxide radicals catalyzed by superoxide dismutase (SOD), and it helps to neutralize the toxicity of phenols and amines [17,18,19]. Additionally, POD exhibits a characteristic absorption peak at 470nm . POD is essential for plants’ adaptation to and resistance against abiotic stresses and is involved in various physiological processes, including growth, development, and senescence. It also contributes to strengthening the plant cell wall through the biopolymerization of hydrophobic lignin precursors.

**Figure 4:**
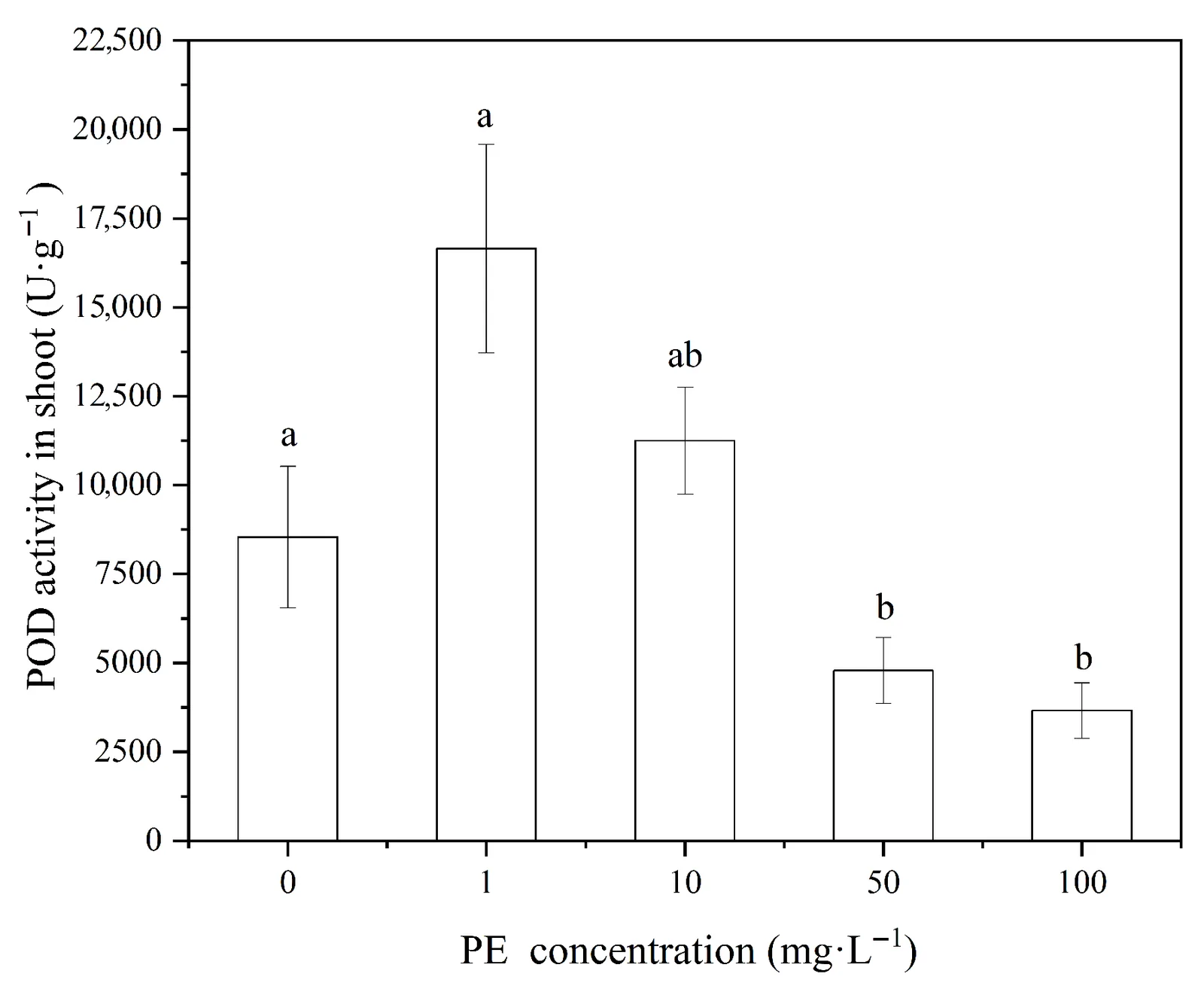
POD activity in wheat shoot at different PE-MPs concentration.

In the current experiment, wheat shoots were exposed to microplastics, which have a uniform particle size of 10μm at different concentrations (1, 10, 50, and $100\;\mathrm{mg}\cdot L^{-1}$ ). This showed varying levels of POD activity from outcome. Specifically, the POD activity in the wheat shoots decreased with increasing concentrations of polyethylene (PE) microplastics, with the activity at $1\;\mathrm{mg}\cdot L^{-1}$ PE being significantly higher than at 10, 50, and $100\;\mathrm{mg}\cdot L^{-1}$ PE ( p<0.05 , Figure 4). Compared to the $0\;\mathrm{mg}\cdot L^{-1}$ PE treatment, lower concentrations of PE ( $1\;\mathrm{mg}\cdot L^{-1}$ ) slightly increased POD activity, while higher concentrations (50 and $100\;\mathrm{mg}\cdot L^{-1}$ ) significantly inhibited POD activity. And Jin et al. found that 6.5μm and 13μm polypropylene (PP) microplastics elevated POD activity in wheat seedlings at high concentrations ( $500\;\mathrm{mg}\cdot {\mathrm{kg}}^{-1}$ ) [20]. Lian et al. also researched a significant increase in POD activity in broad beans (*red*) exposed to 5μm polystyrene (PS) microplastics at concentrations of $10,50,\;\mathrm{and}\;100\;\mathrm{mg}\cdot L^{-1}$ [20]. These studies underscore that the effects of microplastics on POD activity can vary based on particle size and concentration.

**Figure 5:**
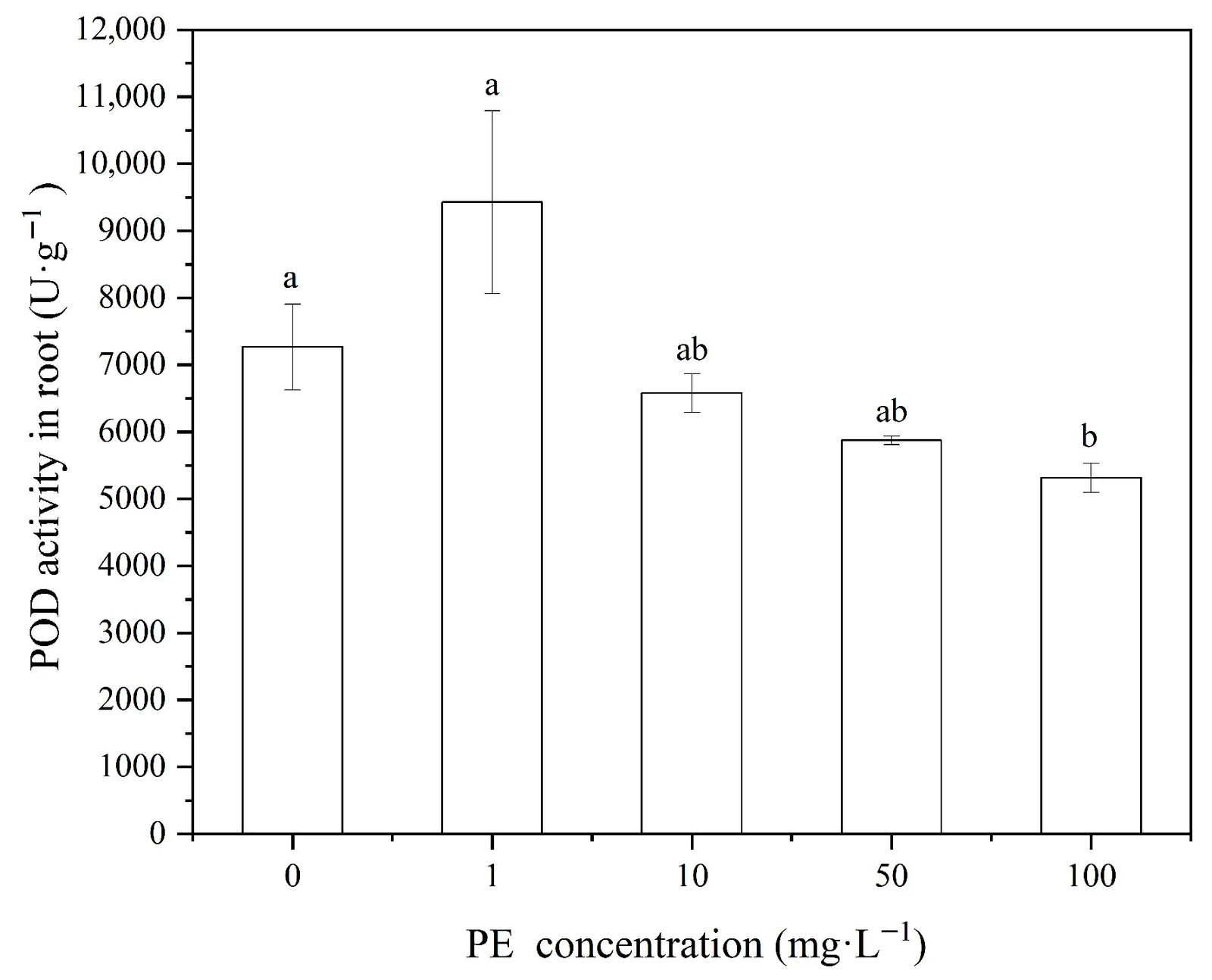
POD activity in wheat shoot at different PE-MPs concentration.

Regarding the wheat root systems, treatment with $1\;\mathrm{mg}\cdot L^{-1}$ PE-MPs did not significantly alter POD activity compared to the control group ( p>0.05 , Figure 5). However, exposure to higher concentrations of PE (10, 50, and $100\;\mathrm{mg}\cdot L^{-1}$ ) significantly inhibited POD activity in the roots ( p<0.05 , Figure 5), which was consistent with the inhibition observed in the shoot system.

In summary, low concentrations of PE suspensions ( $1\;\mathrm{mg}\cdot L^{-1}$ ) did not significantly affect POD activity in wheat roots, but they slightly promoted POD activity in the shoots. On the other hand, high concentrations of PE (50 and $100\;\mathrm{mg}\cdot L^{-1}$ ) significantly inhibited POD activity in both the roots and shoots ( p<0.05 ). Those results indicate a concentration-dependent effect of PE microplastics on POD activity, with higher concentrations inducing significant oxidative stress.

### 3.2. Effects of PE-MPs and ${\mathrm{Cd}}^{2+}$ on Wheat Seedlings

To investigate the effects of combined microplastic and heavy metal pollution on physiological indicators of wheat seedling growth, we conducted a second-phase experiment building upon the first phase to explore and analyze the impacts of combined PE and Cd pollution.

Previous studies have found that higher concentrations of PE significantly affect the POD activities and dry weight in wheat, consistent with the findings of many current studies. Different concentrations of polyethylene (PE-MPs) can exert varying effects on the growth of plants, fish, and other organisms. For instance, Engeseth et al. observed that lettuce exposed to $100\;\mathrm{mg}\cdot L^{-1}$ PE-MPs for 30 days exhibited a 22% reduction in leaf chlorophyll content and a 35% decrease in ascorbic acid (vitamin C) [21]. Wang et al. also reported that $50\;\mathrm{mg}\cdot L^{-1}$ PE microplastics significantly inhibited seed germination, root growth, and dry weight in both soybeans and mung beans, with soybeans exhibiting higher sensitivity to PE than mung beans [22].

However, to avoid excessive PE-MPs masking their toxicity and insufficient concentrations of PE-MPs yielding negligible effects, suspensions were prepared by mixing $50\;\mathrm{mg}\cdot L^{-1}$ PE with Cd solutions at different concentration gradients ( $1,\;5,\;25,\;50\;\mu \mathrm{mol}\cdot L^{-1}$ ) to cultivate wheat seedlings in hydroponics. By analyzing the effects of Cd solutions at different concentration gradients and polyethylene (PE-MPs) on physiological growth indicators of wheat seedlings, the following conclusions were drawn.

#### 3.2.1. Effects of PE-MPs and ${\mathrm{Cd}}^{2+}$ on the Biomass in Wheat Seedlings

In this experiment, the dry weight data for the 5 Cd treatment groups in both the shoots and roots, as well as for the $50\;\mathrm{mg}\cdot L^{-1}$ PE + $0,\;1,\;5,\;25,\;50\;\mu \mathrm{mol}\cdot L^{-1}$ Cd treatment group in the roots, are shown in Table 2. Compared to the control group ( $0\;\mu \mathrm{mol}\cdot L^{-1}\;{\mathrm{Cd}}^{2+}$ ), no significant differences in dry weight were observed in the leaf and root systems of wheat seedlings exposed to different concentrations of ${\mathrm{Cd}}^{2+}$ . However, at low concentrations of ${\mathrm{Cd}}^{2+}$ (1 and $5\;\mu \mathrm{mol}\cdot L^{-1}$ ), the wheat shoots dry weight was reduced compared to the control group, except at higher concentrations (25 and $50\;\mu \mathrm{mol}\cdot L^{-1}$ ), where the dry weight of the shoots exceeded that of the control group. In the root systems, the dry weight was lower than that of the control group at all Cd concentrations, consistent with the results from the PE treatment alone.

In the mixed PE + Cd contamination treatment, no significant differences were found in root biomass compared to the control group.However, in the shoots, the dry weight was observed to increase significantly at the high concentration of ${\mathrm{Cd}}^{2+}$ ( $50\;\mu \mathrm{mol}\cdot L^{-1}$ ), while lower concentrations ( $1,5,\;\mathrm{and}\;25\;\mu \mathrm{mol}\cdot L^{-1}$ ) did not show obvious differences compared to the control group.

These results show that the joint exposure to PE-MPs and ${\mathrm{Cd}}^{2+}$ did not notably influence the biomass of wheat roots and shoots, but did alter the biomass. The results also indicate that the effects of ${\mathrm{Cd}}^{2+}$ on plant biomass may be concentration-dependent, with lower concentrations generally having a less pronounced impact. The dry weight measurement error is subject to significant fluctuations.

#### 3.2.2. Impact of PE-MPs and ${\mathrm{Cd}}^{2+}$ on the Peroxidase Activity in Wheat Seedlings

**Figure 6:**
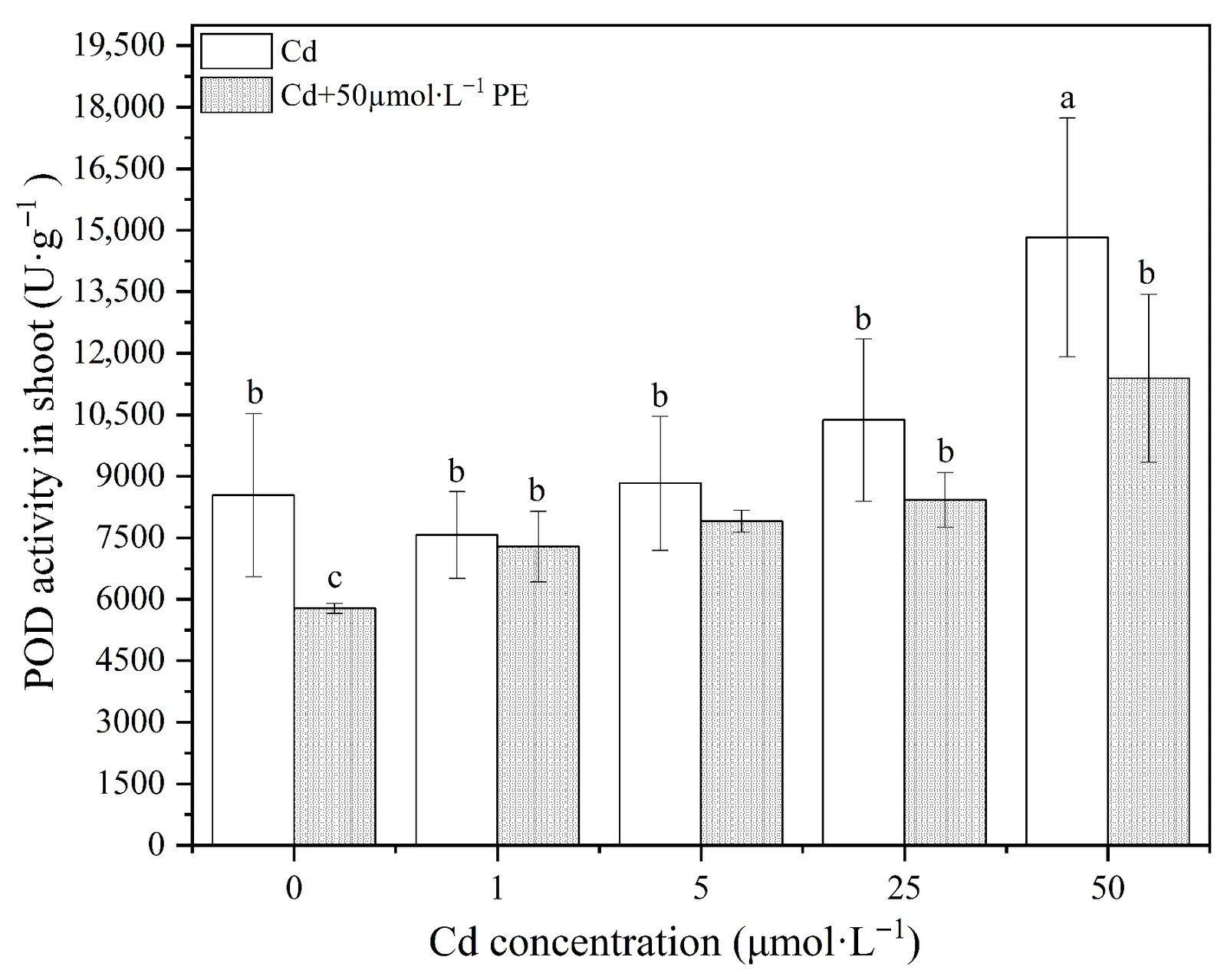
POD activity in wheat shoots under Cd treatments and PE+Cd combined contamination.

When exposed to different concentrations of Cd, the peroxidase (POD) activity in the wheat shoots showed an increasing trend with higher Cd concentrations, with the order of $25\;\mu \mathrm{mol}\cdot L^{-1}>5\;\mu \mathrm{mol}\cdot L^{-1}>0\;\mu \mathrm{mol}\cdot L^{-1}>1\;\mu \mathrm{mol}\cdot L^{-1}$ . Specifically, POD activity in the shoots of wheat seedlings generally increased as the Cd concentration rose, although low concentrations of Cd did not significantly affect POD activity ( p>0.05 , Figure 6). At a high concentration of $50\;\mu \mathrm{mol}\cdot L^{-1}$ Cd, POD activity in the wheat shoots increased significantly by 73.7% compared to the control group (CK, p<0.05 ). This indicates that heavy metal Cd can promote POD activity in wheat shoots, with the effect becoming more pronounced at higher concentrations.It is suggested that under exogenous Cd stress, the reactive oxygen species (ROS) stimulates the upregulation of POD activity due to accumulation, helping the plant mitigate the oxidative stress induced by Cd.

When wheat seedlings were exposed to a combination of $50\;\mathrm{mg}\cdot L^{-1}$ PE and Cd at concentrations ( $50\;\mu \mathrm{mol}\cdot L^{-1}$ ), significant differences in POD activity were observed in the shoots compared to the control group, showing an upward trend with increasing Cd concentration. However, the addition of PE suspension generally reduced POD activity in wheat shoots compared to Cd treatment alone. Specifically, the presence of $50\;\mathrm{mg}\cdot L^{-1}$ PE did not significantly alter POD activity in the wheat shoots ( p>0.05 ), suggesting that PE alone did not significantly activate the antioxidant enzyme system in the shoots. This indicates that the alleviation of Cd toxicity in wheat shoots observed in the combined treatment may not be directly linked to the activation of the antioxidant enzyme system. It is possible that other mechanisms contribute to the reduced cadmium toxicity observed in the plants.

**Figure 7:**
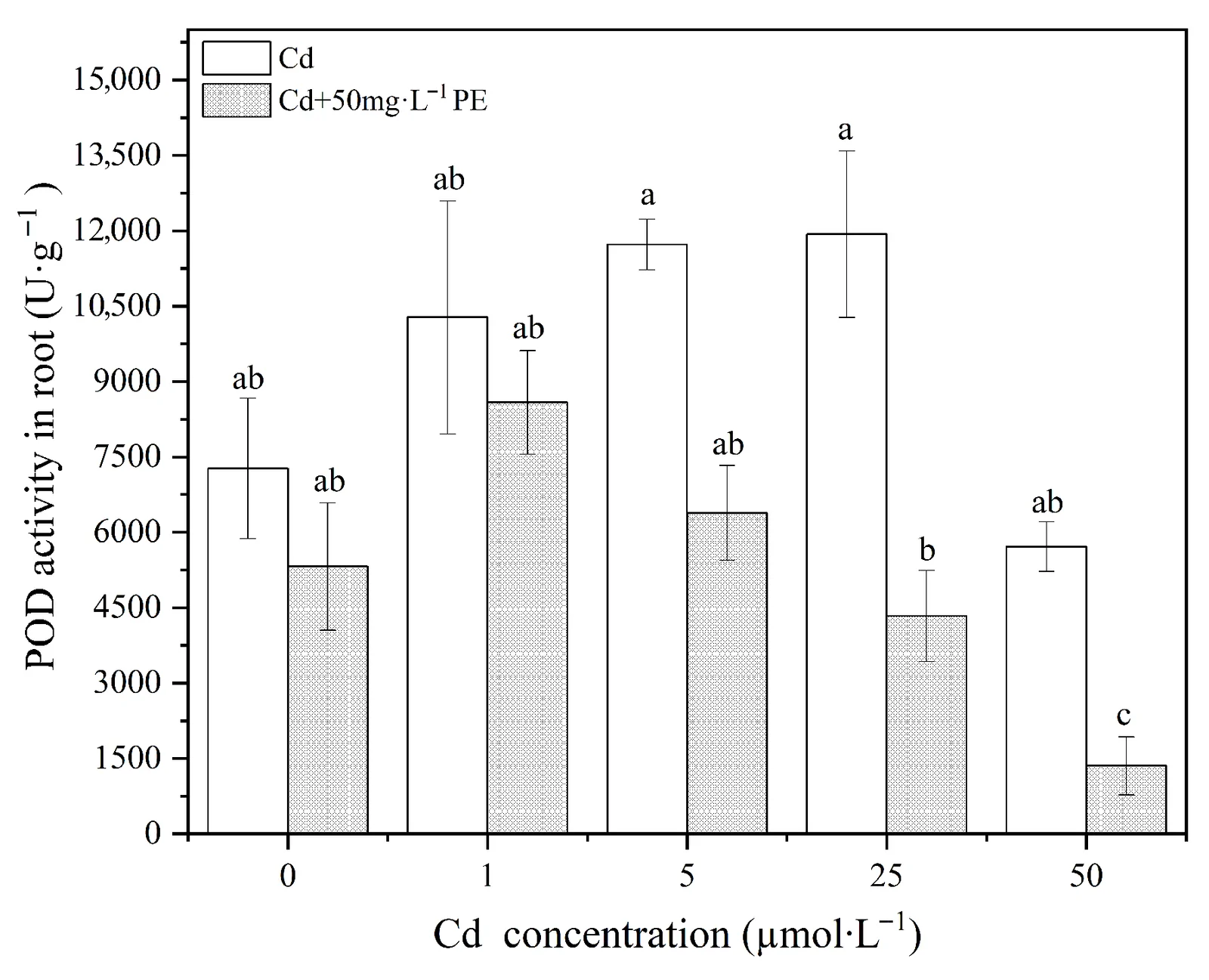
POD activity in wheat root under Cd treatments and PE+Cd combined contamination.

Regarding the wheat roots, exposure to different concentrations of Cd resulted in a general increase in POD activity, with significant differences observed at $5\;\mu \mathrm{mol}\cdot L^{-1}$ and $25\;\mu \mathrm{mol}\cdot L^{-1}$ Cd ( p<0.05 , Figure 7). This increases in POD activity represents a regulatory response by the plant to counteract the environmental stress caused by Cd exposure. However, at the highest concentration of $50\;\mu \mathrm{mol}\cdot L^{-1}$ Cd, a slight decreasing in POD activity was observed, although it was not statistically significant. In the PE+Cd mixed treatments, POD activity in the roots initially increased and then decreased as Cd concentrations rose. A significant difference in POD activity was observed under the $50\;\mu \mathrm{mol}\cdot L^{-1}$ Cd mixed environment. The transient increasing in POD activity at Cd concentrations of $1\;\mu \mathrm{mol}\cdot L^{-1}$ and $5\;\mu \mathrm{mol}\cdot L^{-1}$ represents a response by the plant to mitigate the effects of external stressors. However, the decreasing in POD activity at $25\;\mu \mathrm{mol}\cdot L^{-1}$ and $50\;\mu \mathrm{mol}\cdot L^{-1}$ Cd suggests that the oxidative stress induced by these concentrations exceeded the plant’s self-regulation threshold, leading to damage to the POD enzyme system.

In summary, while low concentrations of Cd promoted POD activity in wheat roots, high concentrations of Cd significantly reduced POD activity in both shoots and roots ( p<0.05 ). The addition of PE ( $50\;\mathrm{mg}\cdot L^{-1}$ ) did not significantly alter POD activity in wheat shoots, suggesting that the interaction between PE and Cd does not solely rely on antioxidant enzyme activation. This implies that other mechanisms may be involved in the reduction of Cd toxicity in wheat.

#### 3.2.3. Impact of PE-MPs on ${\mathrm{Cd}}^{2+}$ Bioaccumulation in Wheat Seedlings

Table 3 presents the Cd accumulation levels in wheat roots and shoots under different treatments. The results indicate that the Cd concentration in wheat shoots significantly increased with higher concentrations of exogenous Cd, primarily due to the increasing lever of Cd in the culture medium. In the Cd-only treatment group, the concentration of Cd in the wheat shoots was consistently higher than in the control group, and the accumulation of Cd increased significantly with rising Cd concentrations. At a Cd concentration of $50\;\mu \mathrm{mol}\cdot L^{-1}$ , the Cd concentration in the wheat shoots reached $129.81\;\mathrm{mg}\cdot {\mathrm{kg}}^{-1}$ .

When PE-MPs concentration of $50\;\mathrm{mg}\cdot L^{-1}$ was combined with different Cd concentration gradients ( $1,\;5,\;25,\;50\;\mu \mathrm{mol}\cdot L^{-1}$ ), the Cd concentration in wheat shoots showed significant changes under composite pollution conditions. Overall, Cd accumulation in the shoots increased with higher Cd concentrations. However, at high concentrations of Cd ( $50\;\mu \mathrm{mol}\cdot L^{-1}$ ), the accumulation plateaued. As the concentration of Cd increases, the addition of $50\;\mathrm{mg}\cdot L^{-1}$ PE significantly enhanced and then decreased Cd accumulation in the shoots. These results suggest that PE microplastics may act as carriers, facilitating the transfer of Cd into wheat shoots, thereby increasing Cd accumulation in environments with low Cd concentrations. However, under high Cd concentrations, PE microplastics may compete with Cd for adsorption sites, leading to reduced Cd accumulation in the shoots. These findings suggest that PE-MPs may mitigate the toxic effects of high Cd concentrations by reducing Cd bioavailability. This aligns with the findings of Jia et al., who demonstrated that microplastics can reduce the bioavailability of Cu and Pb, thereby mitigating their toxic effects on plants [23].

The addition of PE-MPs at various concentrations had almost no effect on Cd accumulation in wheat shoots and roots when no exogenous Cd was added (Table 4). This indicates that PE-MPs alone have minimal impact on Cd accumulation, aggrement with studies where adding PE-MPs did not obviously impact Cd levels in corn and lettuce [24,25]. In the composite pollution treatment groups, the addition of PE-MPs had no significant effect on Cd concentrations in the wheat roots at $1\;\mu \mathrm{mol}\cdot L^{-1}$ Cd. However, at higher concentrations of 25 and $50\;\mu \mathrm{mol}\cdot L^{-1}$ Cd, the Cd concentrations in the roots increased by 162.1% , 18.8% , and 15.5% , respectively. This suggests that $50\;\mathrm{mg}\cdot L^{-1}$ PE enhanced the ability of wheat roots to absorb Cd to some extent. This finding is consistent with that of Xue et al. [26], who reported that the presence of PVC significantly increased Cd concentrations in Arabidopsis roots, resulting in more severe growth inhibition of the plants. Similarly, a study [27] studied the effects of microplastics and Cd contamination on lettuce and found that microplastics promoted Cd uptake by altering soil physicochemical properties. However, this study was conducted under hydroponic conditions, where soil physicochemical properties remained unchanged. Cao et al. [28] reported that under hydroponic conditions, microplastics and co-existing heavy metals interact through electrostatic forces and non-covalent bonds, thus affecting the bioavailability of metal ions. Therefore, the results suggest that PE-MPs may act as a carrier, enhancing Cd bioaccumulation in wheat roots.Other possibilities have also been mentioned in some studies, including reduced gene expression in signaling pathways associated with metals [7].

In summary, based on the above significance analysis results, the mechanisms of cadmium absorption, transport, accumulation, and utilization in wheat seedlings can be categorized into two major aspects. On one hand, environmental stress or microplastic PE may disrupt the expression of cadmium channel genes synthesized by the seedlings themselves, thereby affecting the root system’s capacity for cadmium uptake, transport, accumulation, and utilization. On the other hand, microplastics influence plant cadmium uptake through multiple pathways, including acting as metal carriers and competing with cadmium for binding sites in the root system. And wheat plants exposed to high concentrations of Cd (25 and $50\;\mu \mathrm{mol}\cdot L^{-1}$ ) showed significant effects of $50\;\mathrm{mg}\cdot L^{-1}$ PE-MPs on Cd accumulation in both shoots and roots. However, the interaction trends were different: synergistic in the roots but antagonistic in the shoots.

## 4. Conclusions

This study explored the combined effects of PE-MPs and Cd on antioxidant enzyme activities and Cd bioaccumulation in hydroponic wheat seedlings. The key findings and their implications are summarized as follows:

1. Low concentrations of PE-MPs ( $1\;\mathrm{mg}\cdot L^{-1}$ ) slightly increased peroxidase (POD) activity in wheat shoots, while higher concentrations (50 and $100\;\mathrm{mg}\cdot L^{-1}$ ) significantly inhibited POD activity. These results indicate that PE-MPs induce oxidative stress at higher concentrations.
2. Under combined exposure to PE-MPs and Cd, a significant decrease in POD activity was observed in wheat shoots, particularly at high Cd concentrations ( $50\;\mu \mathrm{mol}\cdot L^{-1}$ ). This suggests that PE-MPs exacerbate the oxidative stress induced by Cd, impairing the plant’s antioxidant defense system.
3. PE-MPs significantly enhanced Cd accumulation in wheat shoots at low Cd concentrations ( $1\;\mu \mathrm{mol}\cdot L^{-1}$ and $5\;\mu \mathrm{mol}\cdot L^{-1}$ ), with Cd concentrations increasing by 150.4% and 51.8% , respectively. However, at high Cd concentrations ( $50\;\mu \mathrm{mol}\cdot L^{-1}$ ), PE-MPs reduced Cd accumulation in shoots but promoted Cd accumulation in roots.

In conclusion, these findings highlight that PE-MPs not only influence Cd bioaccumulation but also alter its toxicity in wheat. At low Cd concentrations, PE-MPs acted as carriers, promoting Cd accumulation in the shoots. In contrast, at high Cd concentrations, PE-MPs reduced Cd accumulation in the shoots by competing for adsorption sites with Cd, while promoting its accumulation in the roots. Due to experimental limitations, this study has constraints in exploring the composite effects of PE-MPs and Cd, preventing an in-depth investigation into the specific pathways through which they influence growth, wheat antioxidant capacity and Cd ion uptake. Future studies should investigate the underlying mechanisms of these interactions and explore their broader ecological impacts on plant health and environmental safety.

## Figures and Tables

**Table 1:** Effect of PE-MPs on the dry weight in wheat seedlings.

Treatments	Dry Weight (g)	
	Root	Shoot
$0\;\mathrm{mg}\cdot L^{-1}$ PE	$0.1507\pm 0.0220^{\;a}$	$0.0606\pm 0.0145^{\;a}$
$1\;\mathrm{mg}\cdot L^{-1}$ PE	$0.1309\pm 0.0329^{\;a}$	$0.0647\pm 0.0059^{\;a}$
$10\;\mathrm{mg}\cdot L^{-1}$ PE	$0.1294\pm 0.0132^{\;a}$	$0.0683\pm 0.0042^{\;a}$
$50\;\mathrm{mg}\cdot L^{-1}$ PE	$0.1210\pm 0.0318^{\;a}$	$0.0603\pm 0.0074^{\;a}$
$100\;\mathrm{mg}\cdot L^{-1}$ PE	$0.1294\pm 0.0064^{\;a}$	$0.0610\pm 0.0102^{\;a}$

Note: The letter “a” indicates no significant differences between treatments.

**Table 2:** Effect of PE + Cd on the dry weight in wheat seedlings.

Treatments	Dry Weight (g)	
	Root	Shoot
$0\;\mu \mathrm{mol}\cdot L^{-1}\;\mathrm{Cd}$	$0.1507\pm 0.0220^{\;\mathrm{ab}}$	$0.0606\pm 0.0145^{\;\mathrm{ab}}$
$1\;\mu \mathrm{mol}\cdot L^{-1}\;\mathrm{Cd}$	$0.1272\pm 0.0149^{\;\mathrm{ab}}$	$0.0585\pm 0.0109^{\;\mathrm{ab}}$
$5\;\mu \mathrm{mol}\cdot L^{-1}\;\mathrm{Cd}$	$0.0975\pm 0.0262^{\;b}$	$0.0512\pm 0.0069^{\;b}$
$25\;\mu \mathrm{mol}\cdot L^{-1}\;\mathrm{Cd}$	$0.1003\pm 0.0145^{\;\mathrm{ab}}$	$0.0633\pm 0.0086^{\;\mathrm{ab}}$
$50\;\mu \mathrm{mol}\cdot L^{-1}\;\mathrm{Cd}$	$0.1251\pm 0.0104^{\;\mathrm{ab}}$	$0.0657\pm 0.0098^{\;\mathrm{ab}}$
$50\;\mathrm{mg}\cdot L^{-1}\;\mathrm{PE}+0\;\mu \mathrm{mol}\cdot L^{-1}\;\mathrm{Cd}$	$0.1478\pm 0.0440^{\;\mathrm{ab}}$	$0.0649\pm 0.0184^{\;\mathrm{ab}}$
$50\;\mathrm{mg}\cdot L^{-1}\;\mathrm{PE}+1\;\mu \mathrm{mol}\cdot L^{-1}\;\mathrm{Cd}$	$0.1592\pm 0.0333^{\;a}$	$0.0616\pm 0.0097^{\;\mathrm{ab}}$
$50\;\mathrm{mg}\cdot L^{-1}\;\mathrm{PE}+5\;\mu \mathrm{mol}\cdot L^{-1}\;\mathrm{Cd}$	$0.1088\pm 0.0301^{\;\mathrm{ab}}$	$0.0563\pm 0.0077^{\;\mathrm{ab}}$
$50\;\mathrm{mg}\cdot L^{-1}\;\mathrm{PE}+25\;\mu \mathrm{mol}\cdot L^{-1}\;\mathrm{Cd}$	$0.1429\pm 0.0404^{\;\mathrm{ab}}$	$0.0572\pm 0.0030^{\;\mathrm{ab}}$
$50\;\mathrm{mg}\cdot L^{-1}\;\mathrm{PE}+50\;\mu \mathrm{mol}\cdot L^{-1}\;\mathrm{Cd}$	$0.1509\pm 0.0147^{\;\mathrm{ab}}$	$0.0777\pm 0.0084^{\;a}$

Note: The letter following the value indicates significance differences ( p<0.05 ) between treatments.

**Table 3:** Cd accumulation in wheat roots and shoots under different treatments.

Treatments	Cd Concentration ( $\mathrm{mg}\cdot {\mathrm{kg}}^{-1}$ )	
	Root	Shoot
$0\;\mu \mathrm{mol}\cdot L^{-1}\;\;\mathrm{Cd}$	$4.42\pm 0.18^{\;f}$	$1.42\pm 0.49^{\;g}$
$1\;\mu \mathrm{mol}\cdot L^{-1}\;\;\mathrm{Cd}$	$8.66\pm 3.33^{\;e}$	$4.46\pm 0.96^{\;f}$
$5\;\mu \mathrm{mol}\cdot L^{-1}\;\;\mathrm{Cd}$	$32.26\pm 12.82^{\;d}$	$28.26\pm 10.66^{\;e}$
$25\;\mu \mathrm{mol}\cdot L^{-1}\;\;\mathrm{Cd}$	$146.59\pm 64.99^{\;c}$	$102.80\pm 46.13^{\;b}$
$50\;\mu \mathrm{mol}\cdot L^{-1}\;\;\mathrm{Cd}$	$191.60\pm 42.56^{\;a}$	$129.81\pm 13.43^{\;a}$
$50\;\mathrm{mg}\cdot L^{-1}\;\mathrm{PE}+0\;\mu \mathrm{mol}\cdot L^{-1}\;\mathrm{Cd}$	$3.61\pm 0.32^{\;f}$	$1.00\pm 0.52^{\;g}$
$50\;\mathrm{mg}\cdot L^{-1}\;\mathrm{PE}+1\;\mu \mathrm{mol}\cdot L^{-1}\;\mathrm{Cd}$	$10.13\pm 2.32^{\;e}$	$4.68\pm 3.84^{\;f}$
$50\;\mathrm{mg}\cdot L^{-1}\;\mathrm{PE}+5\;\mu \mathrm{mol}\cdot L^{-1}\;\mathrm{Cd}$	$32.60\pm 10.87^{\;d}$	$42.89\pm 18.14^{\;d}$
$50\;\mathrm{mg}\cdot L^{-1}\;\mathrm{PE}+25\;\mu \mathrm{mol}\cdot L^{-1}\;\mathrm{Cd}$	$174.19\pm 19.10^{\;b}$	$97.82\pm 53.00^{\;\mathrm{bc}}$
$50\;\mathrm{mg}\cdot L^{-1}\;\mathrm{PE}+50\;\mu \mathrm{mol}\cdot L^{-1}\;\mathrm{Cd}$	$221.36\pm 50.25^{\;a}$	$92.87\pm 49.14^{\;c}$

Note: Different letters indicate significant differences (*p* < 0.05).

**Table 4:** Effect of PE-MPs on Cd concentration in wheat seedlings.

Treatments	Cd Concentration ( $\mathrm{mg}\cdot {\mathrm{kg}}^{-1}$ )	
	Root	Shoot
$0\;\mathrm{mg}\cdot L^{-1}\;\mathrm{PE}$	$4.42\pm 0.18^{\;b}$	$1.42\pm 0.49^{\;b}$
$1\;\mathrm{mg}\cdot L^{-1}\;\mathrm{PE}$	$5.26\pm 2.29^{\;b}$	$2.52\pm 1.50^{\;b}$
$10\;\mathrm{mg}\cdot L^{-1}\;\mathrm{PE}$	$4.83\pm 2.26^{\;b}$	$1.42\pm 0.15^{\;b}$
$50\;\mathrm{mg}\cdot L^{-1}\;\mathrm{PE}$	$25.50\pm 7.80^{\;a}$	$12.89\pm 2.11^{\;a}$
$100\;\mathrm{mg}\cdot L^{-1}\;\mathrm{PE}$	$5.01\pm 0.59^{\;b}$	$2.34\pm 0.45^{\;b}$

Note: Different letters indicate significant differences ( p<0.05 ).

## Data Availability

All related data are provided within the manuscript.
