# Correction: Yang et al. Ecotoxicological Impacts of Microplastics and Cadmium Pollution on Wheat Seedlings. *Nanomaterials* 2026, *16*, 90

**DOI:** 10.3390/nano16211449

**Published:** 2026-11-09

**Authors:** Shuailing Yang, Steven Xu, Tianci Guo, Zhangdong Wei, Xingchen Fan, Shuyu Liang, Lin Wang

**Affiliations:** 1 Miami College, Jinming Campus, Henan University, Kaifeng 475004, China; lyzy8570@163.com (S.Y.); gtc5342@henu.edu.cn (T.G.); 18063388582@163.com (X.F.); 19836887018@163.com (S.L.); 2 Thermo Fischer Scientific-PPD Bioanalytical Laboratory Address, 3230 Deming Way, Middleton, WI 53563-1475, USA; scx1@scarletmail.rutgers.edu; 3 College of Geographical Sciences, Faculty of Geographical Science and Engineering, Henan University, Zhengzhou 450046, China

## Error in Table

In the original publication [1], there was a mistake in Table 4. For the Table 4 Shoot column, 1.42 ± 0.49 ^b^ (the third line) of 10 mg·L^−1^ PE should be changed into 1.42 ± 0.15 ^b^.

The corrected Table 4 appears below:

**Table 4:** Effect of PE-MPs on Cd concentration in wheat seedlings.

Treatments	Cd Concentration (mg · kg^−1^)	
	Root	Shoot
0 mg·L^−1^ PE	4.42 ± 0.18 ^b^	1.42 ± 0.49 ^b^
1 mg·L^−1^ PE	5.26 ± 2.29 ^b^	2.52 ± 1.50 ^b^
10 mg·L^−1^ PE	4.83 ± 2.26 ^b^	1.42 ± 0.15 ^b^
50 mg·L^−1^ PE	25.50 ± 7.80 ^a^	12.89 ± 2.11 ^a^
100 mg·L^−1^ PE	5.01 ± 0.59 ^b^	2.34 ± 0.45 ^b^

Note: Different letters indicate significant differences (*p* < 0.05).

## Text Correction

In Section 2.2.2, one sentence in paragraph one was incorrectly worded and did not accurately convey the intended meaning. 

“Approximately 0.5000 g of each sample was placed into a Teflon digestion tube” should be corrected to “Each dried sample was placed into a Teflon digestion tube”.

The authors state that the scientific conclusions are unaffected. This correction was approved by the Academic Editor. The original publication has also been updated.

## References

[1] Yang S., Xu S., Guo T., Wei Z., Fan X., Liang S., Wang L. (2026). Ecotoxicological Impacts of Microplastics and Cadmium Pollution on Wheat Seedlings. Nanomaterials.

